# Psychological Burden in Female, Iraqi Refugees Who Suffered Extreme Violence by the “Islamic State”: The Perspective of Care Providers

**DOI:** 10.3389/fpsyt.2018.00562

**Published:** 2018-11-08

**Authors:** Caroline Rometsch-Ogioun El Sount, Jana Katharina Denkinger, Petra Windthorst, Christoph Nikendei, David Kindermann, Viola Renner, Johanna Ringwald, Sara Brucker, Virginia M. Tran, Stephan Zipfel, Florian Junne

**Affiliations:** ^1^Department of Psychosomatic Medicine and Psychotherapy, University Hospital Tübingen, Tübingen, Germany; ^2^Department of General Internal Medicine and Psychosomatics, University Hospital Heidelberg, Heidelberg, Germany; ^3^Department of Psychiatry, Psychosomatic Medicine and Psychotherapy for children and Youth, University Hospital Tübingen, Tübingen, Germany; ^4^Department of Gynecology, University Hospital Tübingen, Tubingen, Germany; ^5^Harvard Humanitarian Initiative, Harvard University, Cambridge, MA, United States; ^6^Harvard Medical School, Harvard University, Boston, MA, United States

**Keywords:** refugees, care providers, trauma, PTSD, psychological burden, somatic symptoms, pain, Yazidi

## Abstract

**Introduction:** A large number of refugees suffer from mental disorders such as post-traumatic stress disorder (PTSD). In the context of a special quota project, 1100 Yazidi women from Northern Iraq who had suffered extreme violence by the so-called Islamic State (IS) were brought to Germany to receive specialized treatment. This study aims to investigate the psychological burden and trauma-related symptoms of these female IS-victims from the perspectives of their care providers.

**Material and methods:** Care providers with various professional backgrounds (*N* = 96) were asked to complete a self-developed questionnaire on a Likert-type scale ranging from 1 (very low) to 7 (very high) analyzing the psychological burden and trauma-related symptoms of the IS-traumatized women since their arrival in Germany. We controlled for potential confounders, namely the care providers' personal experiences of trauma and flight, by using chi-square tests.

**Results:** The mean psychological burden for the whole period in Germany as perceived by care providers was *M* = 5.51 (*SD* = 0.94). As the main factors of distress the care providers reported: worries about family members in Iraq (*M* = 6.69; *SD* = 0.69), worries about relatives' possibilities to be granted asylum in Germany (*M* = 6.62; *SD* = 0.68), and uncertainties regarding their future (*M* = 5.89; *SD* = 1.02). The most prominent trauma-related psychological symptoms were nightmares (*M* = 6.43; *SD* = 0.54). The care providers reported that somatic complaints have been present among the refugees in the following manifestation: pain (*M* = 6.24; *SD* = 1.08), gastrointestinal complaints (*M* = 4.62; *SD* = 1.62), and dizziness (*M* = 4.40; *SD* = 1.59). The care providers' personal experiences of trauma and flight had no significant influence on their response behavior.

**Discussion:** Care providers working with IS-traumatized female refugees evaluate the psychological burden and trauma-related somatic and psychological symptom loads of their clients as very high. The results of this study provide important information about the perceptions of care providers working in a refugee-services context and may provide insights for the progression of specialized treatment programs and interventions for highly traumatized refugees and culture-sensitive training programs for their care providers.

## Introduction

According to the United Nations High Commissioner for Refugees (UNHCR), by the middle of September 2018, 68.5 million people were forcibly displaced worldwide. Of this number, 24.5 million are refugees, half of them under the age of 18 years, with still-growing numbers (1). It is estimated that by the year 2015, one million refugees had entered Germany, mainly from Syria, Afghanistan, and Iraq (2), and that by the year 2016, ~745,545 refugees had applied for asylum in Germany (3). The term “refugee” can be defined as a person who was forced to leave his country of origin because of his race, religion, nationality or political beliefs. Returning to their home country is currently impossible for refugees (4, 5). Migration itself may bring about a drastic change in a person's life, but not all migrants respond to these experiences in the same way. Furthermore, not every migrant will demonstrate symptoms of mental illness (6). However, refugees are a more vulnerable group of migrants, with prevalence rates of mental disorders twice as high as those identified among labor migrants (7). There are correlations between the compromised mental health of refugees and increased exposure to violence (8). As Zipfel et al. (9) have described, 10–40% of refugees suffer from mental disorders after experiencing serious traumatic events in their country of origin (10, 11). Moreover, refugees are found to have been exposed to more mental distress than non-refugees (12), causing damage to their mental health. Identified risk factors for developing a mental disorder as a refugee can be found in all three phases of migration: (1). Premigration stress: before fleeing, refugees may face traumatic experiences such as torture and persecution or economic hardships. (2). Migration stress: During migration, refugees may be forced to cope with separations from their families and suffer physical harm. (3). Post-migration stress: Refugees may face complex asylum-seeking processes, language barriers, poor socioeconomic conditions (i.e., unemployment) and acculturation issues (13–16). Above all other mental disorders, high incidences of post-traumatic stress disorder (PTSD) were found among refugees (8). PTSD is a mental disorder that can occur after an individual has experienced or witnessed traumatic events such as natural disasters, serious accidents, terrorist acts, war/combat, rape, or other violent personal assaults. Patients with PTSD may suffer from intrusive thoughts about the traumatic experience and hyperarousal expressed in nervousness, difficulty concentrating or sleep disturbances. Moreover, patients with PTSD tend to avoid situations that remind them of the traumatic event (17, 18).

Existing scientific literature addressing mental disorders is heterogeneous due to methodological issues. However, most studies show significantly higher prevalence of mental disorders among refugees, depending on their country and region of origin (19). Priebe et al. found a higher prevalence of PTSD and depression among refugees compared to the general population of the host country (16). Close et al. (20) reported that the prevalence of PTSD is higher among refugees, with a range from 9 to 36%, compared to the general population with prevalence rates of 1–2% (20). Bozorgmehr et al. identified prevalence rates of 30.6% for PTSD in refugees (11). Furthermore, refugees who have been subjected to torture and/or rape have been found to have the highest PTSD rates (21, 22). Studies of German investigations show a prevalence of PTSD of 23.6% in refugees, with the highest PTSD-prevalence numbers for refugees from Eastern countries (23). For Iraqi refugees with PTSD, the prevalence ranges from 8 to 37.2% (24). Studies focusing on the mental health status of female refugees are rare. In a North Korean female refugee population, significantly higher rates of suicidal ideation and alcohol use were found following sexual violation compared to refugees who had not suffered such traumatic experiences (25).

A large number of studies have also described another important characteristic of traumatized refugees: physical complaints associated with PTSD (26, 27). Hoge et al. (26), as well as Gupta (27), found an association between PTSD and somatic symptoms, including pain occurring in the stomach, head, chest, arms, and legs. Other physical complaints include dizziness, palpitations, shortness of breath, nausea, difficulty sleeping (27), tinnitus, and blurry vision (28). Moreover, there is a well-known high prevalence of comorbidity between PTSD and chronic pain (29).

A group of extremely traumatized women can be found in the community of Yazidi refugees from Northern Iraq. In August 2014, the jihadist terror organization known as the “Islamic State” (IS) attacked the Yazidi community living in the area of Mount Sinjar in Northern Iraq. The Yazidi community has been subjected to killings, enslavement, forced conversion to Islam, systematic rape, and other forms of torture perpetrated by the IS. Yazidi men and young boys have been killed, separated from their families, and forced into IS training camps. Yazidi women and girls have been sold at markets, held in sexual slavery, and forced to marry IS fighters (9, 30–33). The UN estimates that ~6300 Yazidis (3,537 of them women) were abducted (30) and another 3200 Yazidi women and children are still being held in captivity by the IS (1). As a result, the State Ministry of Baden-Württemberg entered into a cooperative arrangement with the Iraqi Kurdish Government and the International Organization of Migration (IOM) to develop an initiative with the aim of ensuring these victims a safe place and the opportunity to receive adequate medical and psychological support in Germany (9, 30). In 2015/2016, a Special Quota Project called the Baden-Württemberg Humanitarian Admission Program (HAP) (30) brought ~1,100 especially vulnerable Yazidi women and children to 22 local districts in Germany (31, 34). The traumatized women receive language courses, education and medical and psychological treatment to enable their long-term integration in Germany. A project with a similar structure is conducted in Canada (35, 36). The women's care providers are mainly from five different professional backgrounds (social workers, interpreters, physicians, psychotherapists and administrators) (9, 30, 34, 37). Due to this highly structured program, the care providers, especially social workers, have the chance to work very closely with each refugee.

Although there are many studies focusing on the psychological and medical burden of refugees in general, e.g., (38, 39), this cross-sectional study aims to explore the specific determinants of psychological burden and medical symptoms of IS-traumatized women from Northern Iraq. However, there are several difficulties in addressing this research question: First, by questioning highly traumatized women, there is a possibility that specific questions serve as a trigger for the participants' trauma contents. This could be a destabilizing experience for the participants and an obstacle in their recovery. Second, for highly traumatized people it can be very hard to concentrate on long questionnaires. The language barrier is an additional issue. Furthermore, the IS-traumatized refugees of the HAP come from the rural area of the Sinjar District, where illiteracy in women is common. A survey using written questionnaires is therefore not feasible in this sample. Moreover, most of the refugees coming from rural areas like the Sinjar District are not familiar with answering questionnaires or research questions, especially when asked to rate medical and psychological symptoms on Likert-scales. Getting meaningful data could therefore pose a challenge.

Following these ethical and practical considerations, we chose to assess the care providers' perspective on the topics in question instead of questioning the refugees directly. Due to their close work with refugees, the care providers are presumed to have deep insights into the psychological burden and trauma-related symptoms from which the refugees suffer. In addition, the perceptions of professional care providers including social workers and psychotherapists with experience in working with refugees from different contexts adds a more objective point of view to the study. The care providers' notion of the refugees' psychological burden, factors of distress and trauma-related symptoms provides implications for treatment and support offers for traumatized refugees as well as for the needs and requirements of both refugees and care providers. Furthermore, an explorative data analysis of the care providers' perspective can build the foundation for addressing the challenge of a specific, hypothesis-driven survey with the refugees themselves.

The purpose of this investigation is to analyze the perspectives of care providers on the psychological burden, factors of distress, and trauma-related symptoms of IS-traumatized female refugees from Northern Iraq. In particular, the study seeks (1) to provide an overview of the care providers' perspective of the overall psychological burden of IS-traumatized women; (2) to identify the specific factors of distress among IS-traumatized women from the care providers' perspective; (3) to analyze the care providers' perception of trauma-related psychological and somatic symptoms of IS-traumatized women.

## Method

### Study design and ethical considerations

This exploratory cross-sectional survey uses self-developed questionnaire-items. The study was conducted in the context of a network meeting of care providers working as part of a Special Quota Project in Baden-Wuerttemberg in April 2017 at the University of Tuebingen. Participants were informed by written information about the study and gave written consent to participate. The questionnaire was administered in the German language. The study was approved by the ethics board (ethic application No. 189/2017BO2) of the University of Tuebingen and fulfills the ethical principles of the Declaration of Helsinki (40).

### Sample

All registered care providers of the HAP (*N* = 132) were invited to participate. *N* = 96 of them completed the questionnaire (response rate = 72.7%). Due to working conditions or illness, not all of the invited care providers were able to participate in the network meeting and the study.

The sample comprises care providers from five different professional backgrounds (social workers, interpreters, physicians, psychotherapists and administrators) working in the context of the HAP with IS-traumatized Yazidi women and children. The care providers were working with varying numbers of women and children at different local sites across Baden-Württemberg, Germany.

### Survey instruments

Sociodemographic data and context characteristics of the care providers and their work in the HAP and were assessed with self-developed questions. Moreover, we asked the care providers specifically if they had trauma or flight experiences themselves.

The care providers' perspective on psychological burden, factors of distress and trauma-related symptoms of IS-traumatized Yazidi women were investigated with a self-developed 7-point-Likert-scaled questionnaire. To the best of our knowledge, no validated questionnaire exists investigating the aim of this study because of the uniqueness of the HAP and this specific refugee group. The used questionnaire can be subdivided into four main topics. First, the care providers' perception of the refugees' current overall psychological burden as well as the mean and maximum overall psychological burden over the whole period the refugees spent in Germany was assessed. Second, the stress-level the care providers perceive in the refugees due to the specific factors of distress shown in Table 1 was identified. Third, the frequency of occurrence of trauma-related (psycho-) somatic symptoms shown in Table 1 was inquired. The forth part of the questionnaire, assessing the trauma-related psychological symptoms depicted in Table 1 was only given to mental health professionals such as doctors and psychotherapists to ensure an objective, professional evaluation regarding these complex symptoms.

**Table 1:** Quantitative questionnaire items regarding psychological burden, factors of distress and trauma-related symptoms of IS-traumatized female refugees, according to care providers' perspectives.

**Questionnaire items with response options using a Likert-type scale ranging from 1 (very low) to 7 (very high)**
•“Please rate the overall psychological burden in the following subunits:”
°“*Current overall psychological burden”*
°“*Mean overall psychological burden over the whole period in Germany”*
°“*Maximum overall psychological burden over the whole period in Germany”*
•“In General, how stressful do you perceive the following factors for the women:”
°“*Ambivalence about returning to the country of origin”*
°“*Illiteracy”*
°“*Fear of hostility of strangers”*
°“*Fear of persecution by ISIS”*
°“*Restrictions of movement”*
°“*limited accessibility of work opportunities”*
°“*Family planning/desire to have children”*
°“*Missing work/tasks”*
°“*Desire to marry”*
°“*Cultural differences”*
°“*Insufficient privacy”*
°“*Differences in religion”*
°“*Language barriers”*
°“*Worries about family members in Iraq”*
°“*Worries that family members cannot follow from Iraq to Germany”*
°“*Social conflicts with beneficiaries”*
°“*Social conflicts with care providers”*
°“*Uncertain future”*
•Trauma related somatic symptoms: “How often do the following symptoms occur in the women in your experience:”
°“*Pain”*
°“*Paresthesia of skin or body”*
°“*Movement disorders”*
°“*Dissociation”*
°“*Functional complaints”*
°“*Feelings of suffocation”*
°“*Dizziness”*
°“*Heart complaints”*
°“*Gastrointestinal complaints”*
•Trauma-related psychological symptoms: “How often do the following symptoms occur in the women in your experience (only answered by mental Health professionals)”:
°“*social withdrawal”*
°“*depression”*
°“*anxiety/panic attacks”*
°“*listlessness”*
°“*agitation (restlessness)”*
°“*nervousness”*
°“*irritability”*
°“*suicidal tendencies”*
°“*self-injury”*
°“*insomnia”*
°“*nightmares”*
°“*flashbacks”*
°“*avoidance of trauma-specific stimuli”*
°“*dissociative disorder”*
°“*eating disorder”*
°“*abuse of medication”*
°“*abuse of alcohol”*
°“*abuse of addictive substances”*
°“*feelings of guilt”*
°“*negative self-image”*

The questionnaire was designed by a team of psychologists and doctors, some of whom have experience working with IS-traumatized Yazidi refugees. According to their expertise and the existing research literature in this field, the most common psychological burden, factors of distress and trauma-related symptoms were included in the survey. To determine the feasibility and appropriateness of this approach and to ensure a correct understanding of the self developed questions, the questionnaire was piloted and adapted by means of expert-interviews with psychologists, social workers and interpreters working in the HAP. The whole questionnaire is available from the authors upon request.

### Data analysis

All quantitative data were analyzed using SPSS [version 24.0.0.1, (41)]. The Psychological burden was analyzed using the mean (*M*) and standard deviation (*SD*) to identify the importance and order of the care providers' ratings of each item. For determining the mean differences, a paired sample *t*-test was used for interval-scaled data. A significance level of α = 0.05 was assumed; for multiple testing, we used the Bonferroni correction of the *p*-value to avoid an accumulation of alpha errors. To investigate the influence of different determinants on the perceptions of care providers chi-squared tests for independence were used 42.

## Results

### Results of the sample description (*N* = 96)

The majority of participants are female (86.5%) with 56.8% of the participants working as social workers. The mean age of the participants is 43.12 years (*SD* = 13.01). On average, the participants already worked in the HAP for 16.3 3 months (*SD* = 6.54). Each care provider was responsible for a mean of 20.39 refugees (*SD* = 27.66). From a mean of 17.1 (*SD* = 13.97) working hours per week in the HAP, the care providers spend 11.1 h (*SD* = 10.84) per week in direct contact with the refugees. Almost a quarter of the care providers (24.0%) reported personal experiences of trauma themselves; 7.3 % reported a personal flight history. Table 2 provides more information regarding the sample description.

**Table 2:** Sample description of the care providers in the Special Quota Project.

**Sample Description**	
**AGE (YEARS)**		
Mean	43.12
SD	13.01
Range	23–66
**GENDER**		
Female	83	86.5%
Male	11	11.5%
**PROFESSION**		
Social education worker/social worker	54	56.8%
Interpreter	11	11.6%
Administrator	11	11.5%
Psychologist/Psychotherapist	6	6.3%
Creative-/Special Therapist	6	6.3%
Therapist for children and youth	3	3.2%
Medical practitioner/Psychiatrist	1	1.1%
Other profession	3	3.1%
**EMPLOYMENT**		
Professional	83	88.3%
Voluntary/training within the profession	2	2.1
Voluntary outside the profession	9	9.6%
**ADDITIONAL TRAINING IN THE TREATMENT OF TRAUMATIZED REFUGEES**		
Yes	39	42.9%
No	52	57.1%
**EXPERIENCES WITH TRAUMATIZED PATIENTS (MONTHS)**		
Mean	69.20
SD	90.57
Range	1–360
**PERSONAL EXPERIENCES OF FLIGHT**		
Yes	7	7.3%
No	83	86.5%
**PERSONAL EXPERIENCES OF TRAUMA**		
Yes	23	24.0%
No	67	69.8%
**PERIOD OF TIME IN THE PROJECT (MONTHS)**		
Mean	16.33
SD	6.54
Range	1–27
**NUMBER OF WOMEN CARED FOR**		
Mean	20.39
SD	27.66
Range	0–111
**WORKING TIME PER WEEK (HOURS)**		
Mean	17.1
SD	13.97
Range	1–48
**DIRECT CONTACT WITH WOMEN (HOURS)**		
Mean	11.11
SD	10.84
Range	0–39

### Psychological burden of IS-traumatized yazidi women

The overall psychological burden of the IS-traumatized women was rated by care providers in three different dimensions: (a) the current overall psychological burden, *M* = 5.29 (*SD* = 0.96), meaning the care providers' perceptions of the refugees' psychological burden during the time this study was conducted; (b) the mean overall psychological burden, *M* = 5.51 (*SD* = 0.94), meaning the care providers' perceptions of the refugees' psychological burden over the entire period that the women resided in Germany; and (c) the maximum overall psychological burden, *M* = 6.04 (*SD* = 1.02), meaning the care providers' perceptions of the refugees' maximum psychological burden during the whole time they resided in Germany.

### Specific factors of distress of IS-traumatized yazidi women

**Figure 1:**
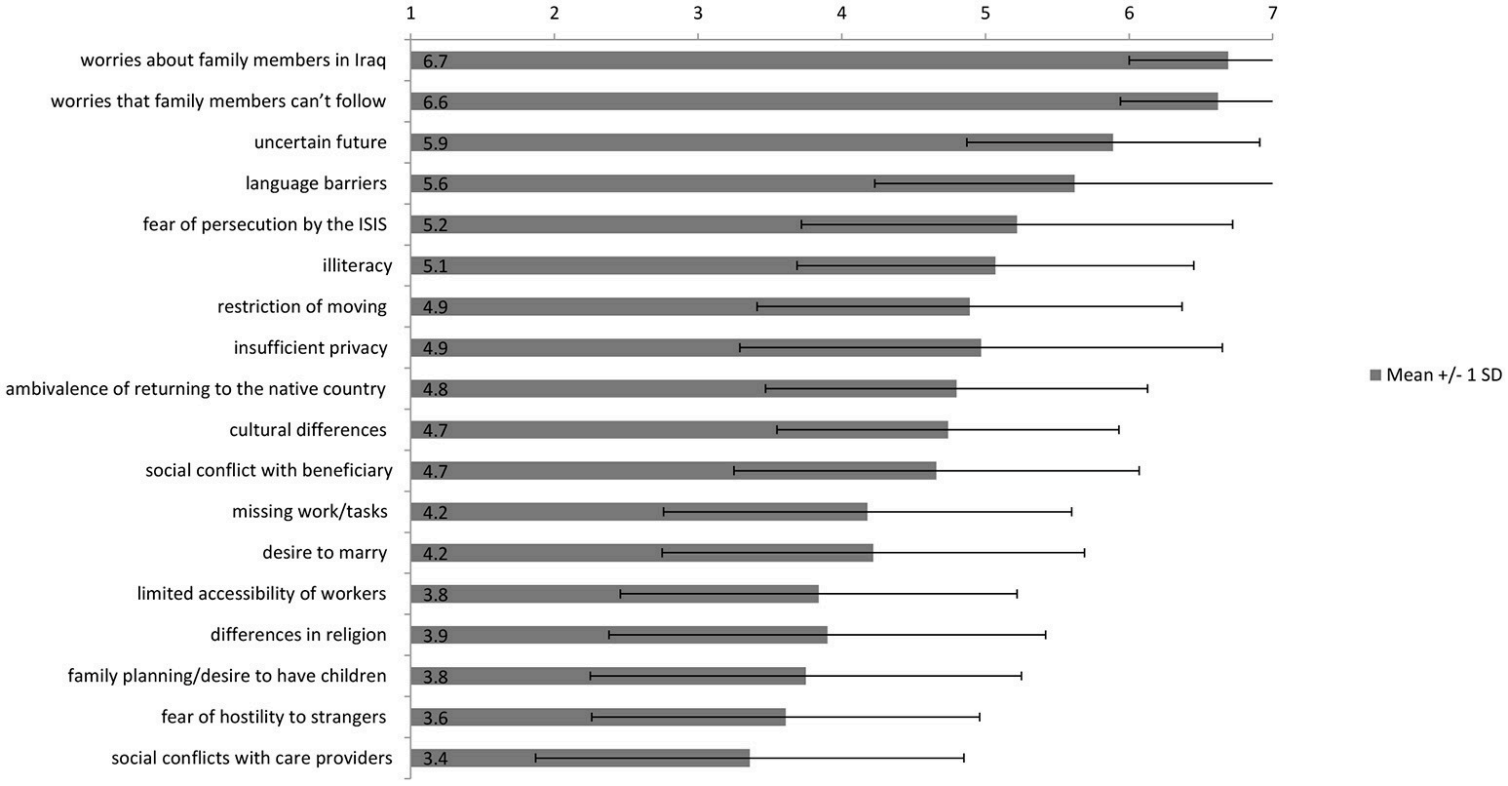
Factors of distress of IS-traumatized refugees according to their care providers. Significance on a level of *p* < 0.000.

The factors the care providers' rated as the most distressing for the refugees were “*worries about family members in Iraq”* (*M* = 6.69, *SD* = 0.69), “*Worries that family members cannot follow (from Iraq to Germany)”* (*M* = 6.62, *SD* = 0.68), and an “*uncertain future”* (*M* = 5.89, *SD* = 1.02). Other factors of distress are illustrated in Figure 1. By calculating paired *t*-tests, it was found that there was no significant difference between the care providers' ratings of “*worries about family members in Iraq”* and “*worries that family members cannot follow (from Iraq to Germany)”, t*_(90)_ = 1.15, *p* = 0.252. However, the care providers rated “*worries about family members in Iraq”* significantly more stressful for the refugees than an “*uncertain future”, t*_(88)_ = 6.72, *p* < 0.000. “*Worries that family members cannot follow (from Iraq to Germany)”* were also rated as a significantly greater burden to the refugees compared to an “*uncertain future”, t*_(88)_ = 6.37, *p* < 0.000. The level of significance using the Bonferroni correction (with 3 items) was α = 0.012.

### Trauma-related symptoms: (psycho-) somatic symptoms of IS-traumatized female refugees

**Figure 2:**
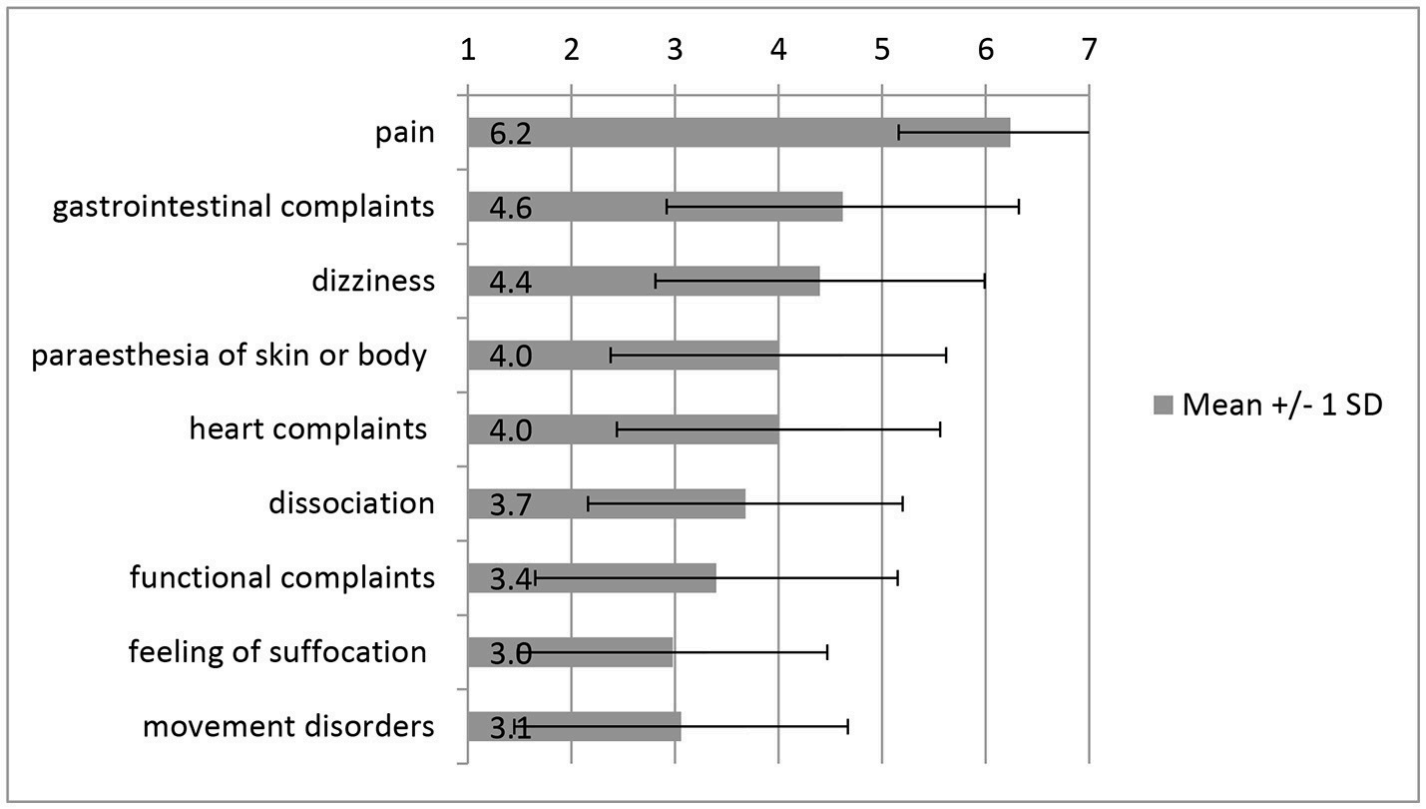
(Psycho-) somatic symptoms of IS-traumatized refugees according to their care providers. Significance on a level of *p* < 0.000.

When asked about the frequency of occurrence of (psycho-)somatic symptoms on the 7-point Likert type scale, the care providers reported “*pain”* (*M* = 6.24, *SD* = 1.08), “*gastrointestinal complaints”* (*M* = 4.62, *SD* = 1.70), and “*dizziness”* (*M* = 4.40, *SD* = 1.59) as the three predominant symptoms. For the ratings of the other symptoms see Figure 2. In free-text answers care providers specified the pain symptoms as “*headaches*” and “*back pain*.” Using a paired *t*-test, it was found that care providers reported “*pain”* as occurring significantly more often in the women than “*gastrointestinal complaints”*, [*t*_(64)_ = 8.23, *p* < 0.000]. “*Pain”* was also rated as occurring significantly more frequent than “*dizziness”*, [*t*_(61)_ = 9.37, *p* < 0.000]. There was no significant difference in the care providers' rating regarding the frequency of occurrence of “*Gastrointestinal complaints”* and “*dizziness”* in the refugees, [*t*_(61)_ = −0.52, *p* = 0.605]. The significance level using the Bonferroni correction (with 3 items) was α = 0.012.

### Trauma-related symptoms: psychological symptoms of IS-traumatized female refugees

According to the mental health professionals' ratings on the above-described Likert-type scale from 1 (“very low”) to 7 (“very high”), the following psychological symptoms are identified as particularly significant: “*nightmares”* (*M* = 6.43, *SD* = 0.54), “*insomnia”* (*M* = 6.43, *SD* = 0.79), and “*depression”* (*M* = 5.71, *SD* = 1.11). However, due to the small number of participating mental health professionals no further statistical analysis was performed with these results. For all ratings see Table 3.

**Table 3:** Psychological burden of IS-traumatized women, according to psychotherapists' perspectives.

**Measure**	
**Psychological burden according to psychotherapists' perspectives**	***M***	***SD***
Nightmares	6.43	0.54
Insomnia	6.43	0.79
Depression	5.71	1.11
Nervousness	5.57	0.54
Avoidance of trauma-specific stimuli	5.57	1.27
Listlessness	5.29	1.25
Anxiety/panic attacks	5.29	1.11
Negative self-image	5.29	1.38
Social withdrawal	5.00	2.08
Irritability	5.14	0.90
Flashbacks	5.14	1.07
Feelings of guilt	5.14	1.77
Agitation (restlessness)	4.71	1.38
Dissociative disorder	3.86	1.35
Abuse of medication	3.00	1.10
Eating disorder	2.86	1.07
Suicidal tendencies	3.29	1.80
Self-injury	2.43	1.40
Abuse of addictive substances	1.67	1.03
Abuse of alcohol	1.71	0.95

### Controlling for potential confounding factors

Since this study assesses psychological burdens of refugees by questioning their care providers, several personal characteristics of the care providers could influence their perception and rating behavior. (43) found that an own trauma or flight history of care providers working with refugees is a significant risk factor for secondary traumatization in care providers (43) which can influence their behavior toward trauma-victims. These personal experiences could also influence the perception of care providers on the refugees and their ratings in this survey. The relation between personal trauma and personal flight experiences and the items “*worries about family members in Iraq,” “worries that family members cannot follow (from Iraq to Germany),”“uncertain future*,” “*pain,” “fears,”* and “*movement restrictions”* was examined using Chi-square tests. The results indicate neither a significant association between “*personal traumatic experiences”* (see Table 4) and the ratings of the included items nor between the care providers' “*personal flight experience”* and their rating behavior (see Table 5). For detecting the determinant of care providers' perceptions of the “*personal flight experience,”* the significance level was adapted according to the Bonferroni correction using multiple testing (5 items) and shows a significance level of α = 0.001.

**Table 4:** Chi-squared test for “*personal trauma experience”* for analyzing any influences in the care providers' ratings of the factors of distress and somatic symptoms of IS-traumatized women.

**Personal trauma experience**		**χ^2^**	***p***	**Cramer's V**
1.	“*worries about family members in Iraq”*	χ^2^ (4, *n* = 85) = 0.78	0.941	0.096
2.	“*worries that family cannot follow”*	χ^2^ (3, *n* = 85) = 3.70	0.296	0.209
3.	“*uncertain future”*	χ^2^ (3, *n* = 83) = 2.66	0.447	0.179
4.	“*pain”*	χ^2^ (6, *n* = 61) = 6.09	0.413	0.316
5.	“*fear of persecution by ISIS”*	χ^2^ (5, *n* = 82) = 1.63	0.898	0.141
6.	“*fear of hostility by strangers”*	χ^2^ (5, *n* = 83) = 2.96	0.706	0.189
7.	“*restriction of movement”*	χ^2^ (6, *n* = 83) = 7.47	0.279	0.300

**Table 5:** Chi-squared test for personal flight experience for analyzing any influences in the care providers' ratings of the factors of distress and somatic symptoms of IS-traumatized women.

**Personal flight experience**		**χ^2^**	***p***	**Cramer's V**
1.	“*worries about family members in Iraq”*	χ^2^ (4, *n* = 85) = 1.38	0.848	0.127
2.	“*worries that family cannot follow”*	χ^2^ (3, *n* = 85) = 4.12	0.249	0.220
3.	“*uncertain future”*	χ^2^ (3, *n* = 83) = 3.01	0.391	0.190
4.	“*fear of persecution by ISIS”*	χ^2^ (5, *n* = 82) = 6.71	0.243	0.286
5.	“*Fear of hostility to strangers”*	χ^2^ (5, *n* = 83) = 6.33	0.276	0.276

## Discussion

To the best of our knowledge, this is the first study to explore the psychological burden, factors of distress, and trauma-related symptoms in a large sample of IS-traumatized females from Northern Iraq from the perspectives of their care providers. The results show that care providers perceive female IS-victims who came to Germany in 2015/2016 as a highly psychologically burdened group with an especially high trauma-related symptom load.

The care providers registered high mean values of psychological burden and distress levels in the refugees during the whole time the refugees resided in Germany. However, there is some variability in the refugees' overall psychological burden rated by their care providers. At the time this survey took place, which was 1–2 years after the refugees first arrived in Germany, the care provider rated the current overall psychological burden of the refugees as less stressful than their mean or maximal psychological burden during their time in Germany. This result suggests that the early post-migration phase is especially stressful for refugees.

Worries about their family members in Iraq and worries that family members will not be granted asylum in Germany are seen as the highest stressors for IS-traumatized women by their care providers. This finding is in line with the recent literature. In an investigation of Iraqi refugees resettled in the US, Gangamma et al. also described the refugees' worries about relatives and personal safety. They found this psychological burden to be omnipresent, even though the refugees themselves did currently not face any threat (44). Other studies confirmed the significant association between family separation and serious mental health problems such as subsyndromal depression, anxiety, or PTSD (45). Intrusive fears about family members are thereby related to greater mental health impairment and current living difficulties (46). This is especially relevant in the context of the refugees in this study. Many of their family members are still held in IS-captivity or live in refugee camps in Iraq (5). Furthermore, due to restrictions in German law, there are no clear prospects for families to reunite in Germany.

Another main factor of distress identified in this study is the uncertainty regarding the refugees' future. This is in line with other studies. El-Shaarawi, for example, described the effects of uncertainty on the mental health of Iraqi refugees in Egypt as well. The author presented evidence for causes of instability leading to distress and restrictions of the refugees' well-being (47).

Regarding trauma-related psychological symptoms, the mental health professionals perceive a wide range of different symptoms as frequently occurring. From the perspectives of mental health professionals, the refugees in this study suffer predominantly from sleeping disorders such as nightmares and insomnia. In addition, specific PTSD-symptoms, e.g., nervousness, avoidance of trauma-related stimuli, listlessness and flashbacks are also rated as occurring often in the refugee sample. Furthermore, the mental health professionals rated affective burdens such as depressive symptoms, anxiety and panic attacks as occurring frequently. In contrast, abuse of alcohol and addictive substances were rarely perceived. Similar symptoms have been found in other studies as well. A study examining Yazidi refugees resettled in Turkey has reported disorders such as PTSD, depression, and anxiety generally as the most often noted mental illnesses among children (48).

The most frequently reported trauma and stress-related symptoms of IS-traumatized females from the perspective of their care providers were pain, gastrointestinal complaints and dizziness. Pain symptoms showed a significantly higher occurrence-rating compared to other somatic symptoms. Pain may become a chronic disease of importance among traumatized refugees (49) with high comorbidity with depression. In the population of Iraqi refugees, pain, particularly back pain, was diagnosed most often (50). Rohlof et al. found more unexplained somatic symptoms in traumatized refugees than in the general Western population (51). Jamil et al. listed next to self-reported somatic complaints medical conditions among Iraqi refugees and found dizziness and gastrointestinal symptoms to be the main health complaints (19, 52). Given these findings, refugees should receive specialized medical support for these somatic symptoms, always with consideration of the psychological load and the traumatic experiences they have suffered. Indeed, ways of expressing psychosocial distress depend on a culture's values, norms, and stereotypes (53). In addition, the personal meaning of having a trauma disorder influences the patient's way of expressing trauma symptoms and his or her help-seeking processes (54). For the successful integration of refugees, cultural sensitivity training for care providers seems to be essential. They need to have some understanding about the refugees' cultural idioms of distress explanatory models, coping strategies, and norms for seeking help (55, 56). Therefore, refugee mental health care should be conducted by an interdisciplinary and multicultural team with knowledge of both trauma symptoms and the cultural values and norms of the Yazidi society.

## Limitations

Using the care providers' perspective introduces potential underlying biases to the study's results. There could have been an overestimation of observable symptoms such as social withdrawal or an underestimation of non-observable symptoms such as nightmares, a negative self-image or feelings of guilt. Interpreting these results, the reader has to keep in mind that the care providers could only answer the questions based on the symptoms they perceived in the refugees or based on what the refugees had told them. However, since the care providers spend on average 11 h per week in direct contact with the refugees, their level of being informed about the psychological burden of the women is assumed to be sufficient. Moreover, specific characteristics of the caregivers could have influenced their ratings. We were able to partly control for this bias by showing that the care providers' response behaviors were not influenced by their own traumatic or flight experiences. Another bias could have occurred due to the different working conditions of the care providers, their different contact durations with the traumatized women and the wide range of the number of refugees they work with. Due to the trauma experienced by Yazidi women, a direct investigation of their burden and symptoms was ethically not justifiable at the time in which the study was conducted. More time was needed to ensure their medical and psychiatric treatment and integration in Germany. Nevertheless, in the future research, a survey asking IS-traumatized female refugees from Northern Iraq about their psychological burdens directly is needed to revise the results of the present study. This could be especially interesting in order to assess in which topics care providers' and refugees' rating match and to which questions they have different answers.

Moreover, because a self-developed questionnaire was used in order to accurately address the characteristics of this specific refugee population, there are no psychometric properties available. It must further be considered that the sample population comprised a special group of refugees, and to what extend the results can be generalized must be accurately determined.

## Conclusion

This study is the first to provide survey data on the psychological burden, factors of distress and trauma-related psychological and somatic symptoms of IS-traumatized women, in their care providers' perspectives. The most important distressing factors, as seen by the care providers, are the women's worries about relatives and their lack of certainty and stability regarding their future. Pain, gastrointestinal complaints and dizziness the care providers considered to be the main somatic-symptom complaints among IS-traumatized female refugees. Care providers' personal trauma and flight experiences do not seem to influence their perceptions of psychological distress or trauma-related symptoms. These findings have important implications for the needs and treatment options of traumatized refugees as well as the necessity for culture-sensitive training programs preparing care providers to work with highly burdened refugees.

## Data availability statement

The raw data supporting the conclusions of this manuscript will be made available by the authors, without undue reservations, to any qualified researcher.

## Author contributions

CR-OES, JD, PW, and FJ planned and conducted the study. CR-OES analyzed the data and wrote the manuscript with support from FJ. JD, PW, CN, DK, SB, VR, JR, VMT and SZ supported the study and the writing process with ideas and feedback.

### Conflict of interest statement

The authors declare that the research was conducted in the absence of any commercial or financial relationships that could be construed as a potential conflict of interest.
